# Length of stay and mean cost of patients’ hospitalization with healthcare-associated infections acquired in a national hospital in Senegal

**DOI:** 10.1186/1753-6561-5-S6-O17

**Published:** 2011-06-29

**Authors:** A Ndir, A Cisse, LP Nadiele, NM Dia Badiane, B Ndoye

**Affiliations:** 1 PRONALIN, Dakar, Senegal; 2 DES MINISTERE DE LA SANTE, Dakar, Senegal; 3 HPD, Dakar, Senegal; 4 CHNU FANN, Dakar, Senegal

## Introduction / objectives

Economic data on healthcare-associated infections (HCAI) in resource-poor countries are practically nonexistent despite the importance of this issue. Healthcare costs are patients’ charges whereas minimum wages is of 72€ in Senegal. The objective of our study was to estimate the length of stay (LOS) and the mean cost of stays of patients who have acquired a HCAI at *Hôpital Principal de Dakar* to increase the awareness of authorities on this issue.

## Methods

Retrospective analysis of the hospital stays' database between September and November 2010. Patients admitted in surgical unit, internal medicine unit and intensive care unit and for who at least a bacteriological test was found positive were selected. Probable cases of HCAI were included on clinical data as the delay of appearance, clinical and bacteriological signs like the presence of multiresistant bacteria. Hospital stay's cost was estimated by the daily cost, the cost of therapeutic acts and biological tests.

## Results

19 cases of HCAI were identified in 16 patients. The LOS is of 56 days for HCAI identified in surgical unit (SU), 39 days in internal medicine unit (IMU) and 20 days in intensive care unit (ICU). The mean cost of these patients’ stays were of 2 793€ in SU, 1 734€ in IMU and 1 578€ in ICU.

## Conclusion

Our study is not exhaustive since infections with no bacteriological data and viral and fungal infections were not included. However, results highlight the economic burden of HCAI. A case-control study would show the overcost induced by HCAI and results would give decisive argument for the prevention of these infections.

## Disclosure of interest

None declared.

## Note

This abstract was also presented as Poster P351.

